# Berberine Inhibits Pro-inflammatory Cytokine-induced IL-6 and CCL11 Production via Modulation of STAT6 Pathway in Human Bronchial Epithelial Cells

**DOI:** 10.7150/ijms.45400

**Published:** 2020-06-08

**Authors:** Jason Ma, Cheng-Chi Chan, Wen-Chung Huang, Ming-Ling Kuo

**Affiliations:** 1 Department of Microbiology and Immunology, Graduate Institute of Biomedical Sciences, College of Medicine, Chang Gung University, Taoyuan, Taiwan.; 2 Cancer Vaccine and Immune Cell Therapy Core Laboratory, Chang Gung Immunology Consortium, Chang Gung Memorial Hospital, Linkou, Taoyuan, Taiwan.; 3 Graduate Institute of Health Industry Technology, Research Center for Food and Cosmetic Safety, College of Human Ecology, Chang Gung University of Science and Technology, Taoyuan City, Taiwan.; 4 Research Center for Chinese Herbal Medicine, College of Human Ecology, Chang Gung University of Science and Technology, Taoyuan City, Taiwan.; 5 Division of Allergy, Asthma, and Rheumatology, Department of Pediatrics, Chang Gung Memorial Hospital, Taoyuan, Taiwan.

**Keywords:** Berberine, cytokines, asthma, eotaxin, epithelial cell

## Abstract

Berberine is an isoquinoline alkaloid isolated from various Chinese herbs that has potential of anti-inflammatory, anti-lipidemic, anti-neoplastic, and anti-diabetic activity. In this study, we evaluated the anti-inflammatory efficacy of berberine on allergic airway inflammation by targeting epithelial cells. Allergic airway inflammation driven by T helper 2 (Th2)-type immunity is characterized by airway hyperresponsiveness, elevated IgE production, and eosinophilic infiltration. For eosinophil recruitment, major chemoattractant CCL11 (eotaxin-1) was secreted by lung epithelial cells. BEAS-2B cells, a human bronchial epithelial cell line, were pre-treated with berberine and then activated by IL-4 plus TNF-α. The viability of BEAS-2B cells was assessed. Expression levels of IL-6 and CCL11 were determined using ELISA and real-time PCR. The signaling pathways of MAP kinases, NF-κB, and STAT6 were analyzed by western blot. Berberine treatment (≤1 μM) didn't significantly affect the viability of BEAS-2B cells with or without IL-4 plus TNF-stimulation. Berberine significantly inhibited the secretion of IL-6 and CCL11 from pro-inflammatory cytokine-activated BEAS-2B cells. NF-κB and MAP kinase pathways were seemingly unaffected in BEAS-2B cells with berberine treatment. Significant reduction of nuclear STAT6 protein expression in activated BEAS-2B cells with berberine treatment was observed. Current study reveals that berberine has inhibitory effect in pro-inflammatory cytokine-activated BEAS-2B cells through reducing IL-6 and CCL11 production, which is possibly modulated by suppressing STAT6 signaling pathway.

## Introduction

Asthma is one of the major public health concerns worldwide, with high prevalence and economic burden 1. Asthma is a chronic inflammatory airway disease characterized by airflow obstruction, persistent inflammation, and airway hyperresponsiveness (AHR) to common environmental aero-allergens 2. Asthmatic patients exhibit elevated allergen-specific IgE, mucus hypersecretion, and eosinophilia in the lungs. Most treatments for asthma are based on western medicine, such as β2-adrenergic receptor agonists, anti-IgE blockers, and corticosteroids combined with a long-acting bronchodilator, but only provide temporary alleviation of the symptoms 3. In addition, steroids have certain side effects and some patients do not favorably respond to this treatment. Nevertheless, traditional Chinese medicines are reported to have improved or curative effects in clinical trials for allergic diseases including asthma 4.

Airway epithelium acts as the first defense barrier against environmental stimuli by secreting numerous chemokines, cytokines and growth factors to recruit and activate a variety of immune cells including CD4^+^ T helper 2 (Th2) cells, mast cells, and eosinophils 5. Th2 cytokines, especially interleukin (IL)-4, IL-5, and IL-13, and other inflammatory mediators secreted by eosinophils and mast cells are primarily thought to orchestrate pathological features of asthma 6. CCL11 (eotaxin-1), the most potent chemokine for eosinophil movement, is secreted by bronchial epithelial and endothelial cells and its receptor, CCR3, is highly expressed on eosinophils 7. CCL11 is also verified to activate eosinophils by eliciting the release of RNase and cell-free granules 8. Significant increase of CCL11 levels is detected in blood, sputum, and exhaled breath condensate (EBC) of asthmatic patients compared with healthy subjects 9. Moreover, the disruption of CCL11 gene expression effectively reduces tissue eosinophilia 10. Therefore, the reduction of eosinophil recruitment by inhibiting CCL11 expression in the lungs might have therapeutic potential for asthma.

The level of pro-inflammatory cytokine IL-6 was elevated in asthma patients 11. A large variety of cells can produce IL-6, including cells from innate immune system, endothelial cells, fibroblasts, epithelial cells, etc. 12. IL-6 functions as a growth factor for B cells and also contributes to CD4 T cells differentiation 13. The presence of IL-6 promotes autocrine IL-4 production by activating STAT3 and ERK pathway 14, IL-5 production by p38 pathway, with the help of IL-2 signaling 15, and further enhances Th2 differentiation through an auto-feedback loop. Nonetheless, elevation of IL-6 is also correlated with elevated level of IL-13 and increased in asthma patients 16. Taken together, targeting IL-6 may alleviate asthmatic responses by obstructing Th2 differentiation.

Previous papers showed that IL-4 and tumor necrosis factor (TNF)-α can stimulate the fibroblasts of nasal polyps to produce eotaxin-1 17, 18. Similarly, other evidences reveal that IL-4 plus TNF-α triggers the eotaxin secretion of human bronchial epithelial cells, BEAS-2B 19. The activation of eotaxin gene expression is regulated by NF-κB and STAT6 in human airway epithelial cells upon IL-4 activation 19. NF-κB pathway is also involved in regulating diverse inflammatory cytokine genes including IL-1β and TNF-α that re-stimulate immune cells and epithelial cells to exacerbate the inflammation 20. With TNF-α, MAP kinases, which comprise the extracellular signal-regulated kinases (ERK), the p38 MAP kinases, and the c-Jun NH2-terminal kinases (JNK), can also be activated 21. MAP kinase signaling pathways transduce a variety of extracellular signals that mediate cellular functions implicated in proliferation, differentiation, inflammation, and apoptosis 22.

Previous studies have reported that berberine, an alkaloid extracted from various traditional Chinese herbs, has pharmacological effects on intestinal bacteria, parasites, cardiovascular and cerebrovascular diseases, lowering blood sugar, and cancer 23. In respiratory tract infections, berberine has been confirmed to suppress inflammatory agents, such as IL-1β and TNF-α by inhibiting IκB degradation in human lung cells 24. Several papers also indicate that berberine decreases the inflammatory response by repressing the inflammation cytokine expression in colonic macrophages and epithelial cells or retinal pigment epithelial cells by down-regulating transcription factor NF-κB or the MAP kinase pathways 25, 26. Based on these evidences, berberine is seemly capable of suppressing the inflammatory responses.

In present study, we applied BEAS-2B cells, a human bronchial epithelial cell line, as an *in vitro* model to examine the anti-inflammatory efficacy of berberine on pro-inflammatory cytokine-stimulated epithelial cells. NF-κB, STAT6 and MAP kinases signaling pathways involved in modulating eotaxin gene expression are assessed.

## Materials and Methods

### Materials

**Figure 1:**
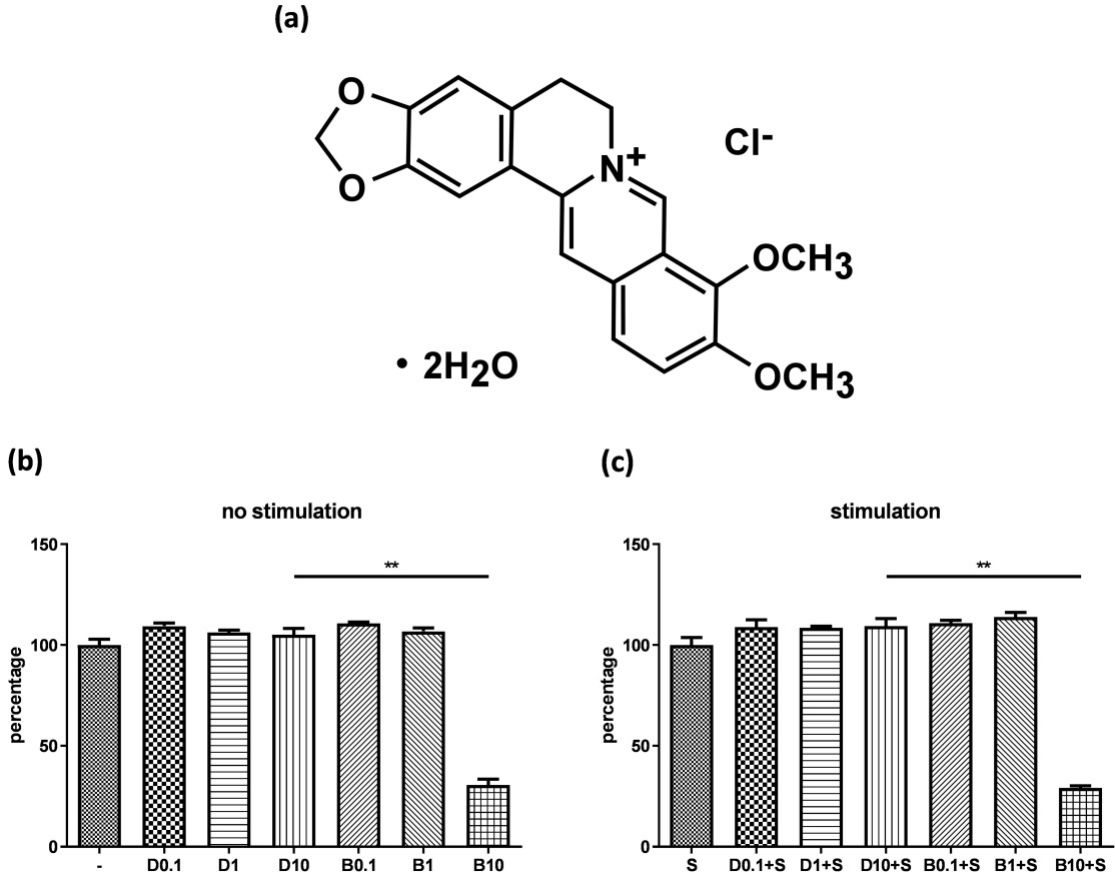
** Cytotoxicity of berberine on human bronchial epithelial cell line.** (A) The chemical structure of berberine. BEAS-2B cells were cultured in the 48-well plates overnight and then were treated with different concentrations of berberine (0.1 μM to 10 μM) or equal volume of DMSO for 16 to 18 hours. Subsequently, drug pre-treated cells were stimulated (A) without or (B) with pro-inflammation cytokines for 24 hours. The cell viability was analyzed by CCK-8 assay. The percentage was calculated by comparing the O.D. value with cell only group. Data are presented as mean ± SEM (n= 6). S, pro-inflammation cytokine stimulation; D, DMSO; B, berberine. The number indicated the concentration (μM) of berberine or DMSO. **P<0.01.

Figure 1A shows the chemical structure of berberine chloride (≥98% purity by TLC; Sigma-Aldrich). A stock solution of 20 mM berberine was prepared in DMSO (Sigma-Aldrich). The final DMSO concentration did not exceed 0.1% in the culture medium.

### Cell culture and berberine treatment

Human bronchial epithelial cells (BEAS-2B CRL-9609 cell line, ATCC) were cultured in Dulbecco's Modified Eagle Medium/Nutrient mixture F-12 (DMEM/F12) medium (Gibco) containing 10% fetal bovine serum (FBS, Gibco) and 1% penicillin/streptomycin (Gibco). Cells were seeded in 6-well plates (2 × 10^6^ cells) or 24-well plates (4x10^5^ cells) at 37 °C in a humidified 5% CO_2_ atmosphere. Berberine chloride (Sigma-Aldrich) was dissolved in DMSO (Sigma-Aldrich), where same amount of DMSO for dissolving berberine was used as control for comparison. BEAS-2B cells were treated with or without different concentrations of berberine for 16-18 hours. After pre-treatment, BEAS-2B cells were stimulated with IL-4 (20 ng/ml) (Peprotech) plus TNF-α (5 ng/ml) (Peprotech) and culture supernatants were harvested after 6 hours and 24 hours to detect IL-6 and CCL11 levels by ELISA assay. For inhibition assays, 10 μM of JNK inhibitor-SP600125 (Enzo) and ERK inhibitor-PD-98059 (Enzo) were added one hour before cytokine stimulation and culture supernatants were harvested after 24 hours for IL-6 and CCL11 detection.

### Cell viability assay

BEAS-2B cells (2×10^5^ cells) were seeded in a 48-well plate overnight and then were pre-treated with various concentrations of berberine or DMSO for 16-18 hours. After pre-treatment, cells were stimulated without or with IL-4 and TNF-α for 24 hours and the viability was analyzed by Cell Counting-kit 8 (CCK-8) (Sigma-Aldrich). Briefly, after the supernatants were harvested, cells were washed with 1 ml PBS and then CCK-8 was added into each well to incubate at 37 °C in a humidified 5% CO_2_ atmosphere for 4 hours. Supernatants were transferred into a 96-well plate after incubation. Absorbance was measured on an ELISA reader at 450 nm (SpectraMax).

### Enzyme-linked immunosorbent assay

Culture supernatants were harvested following 6 or 24 hours of IL-4 and TNF-α stimulation. The levels of IL-6 and CCL11 (DuoSet ELISA kit, R&D systems) were determined by ELISA according to the manufacturer's instructions.

### RNA isolation and real-time polymerase chain reaction

RNA samples were collected at 3, 6, 12 and 24 hours after cytokine stimulation. Total RNA was extracted using TRIzol reagent (Invitrogen). Reverse transcription was performed by oligo-dT primers and M-MLV reverse transcriptase (Invitrogen). The mRNA expression of GAPDH, IL-6, and CCL11 were amplified by specific primers: 5'-GCAAATTCCATGGCACCG-3' for GAPDH forward primer and 5'-TCGCCCCACTGATTTTGG-3' for GAPDH reverse primer; 5'-CCAATCTGGATTCAATGAGGAG-3' for IL-6 forward primer and 5'-GGTCAGGGGTGGTTATTGCATC-3' for IL-6 reverse primer; 5'-AAAGCTCACACCTTCAGCCT-3' for CCL11 forward primer and 5'-TTTCTGGGGACATTTGCCAC-3' for CCL11 reverse primer. For real-time PCR, the reaction was performed by Lightcycler® 96 system (Roche) with iQ^TM^ SYBR green supermix (Bio-rad). The relative expression of each gene was calculated by normalizing to the expression of GAPDH.

### The extraction of total cell, cytoplasm and nucleus protein

For total cell protein extraction, protein samples were collected at indicated time point after cytokine stimulation. Cells were lysed in cell lysis buffer containing 1 mM Na_3_VO_4_, 1 mM Tetranisole, 25X diluted protease inhibitor, 5 μM MgCl_2_, 20 μM Tris-HCl, 50 μM NaCl, 20% NP-40 on ice for 30 min. Cell lysates were centrifuged at 12,000 g for 30 min at 4 °C and the supernatants were harvested for western blot analysis. For cytoplasm and nucleus protein extraction, cell extracts were collected using Nuclear and Cytoplasmic Extraction Reagent kit (NE-PER kit) (Pierce Biotechnology) according to the manufacturer's instructions. Protein concentrations were determined using a BCA protein assay (Pierce Biotechnology) for western blot analysis.

### Western blot analysis

Protein samples (20 μg) were separated on sodium dodecyl sulfate-polyacrylamide gel, transferred to nitrocellulose membrane (Millipore) at 50 mA for 30 min and blocked in Tris-buffered saline with 0.1% Tween 20 (TBST) containing 5% BSA at room temperature for 1 hour. The blots were incubated with primary antibodies (rabbit anti-human antibody) to JNK, phosphorylated JNK, ERK, phosphorylated ERK, p38, phosphorylated p38, IκB, phosphorylated IκB, p65, STAT6, and phosphorylated STAT6, primary antibodies (mouse anti-human antibody) for β-actin and p38, and primary antibodies (goat anti-human antibody) for PCNA (Cell Signaling) overnight at 4 °C. Secondary antibodies conjugated with horseradish peroxidase were incubated for 2 hours. Finally, the blots were developed by using the Immobilon Western chemiluminescent HRP substrate (Millipore).

### Statistical analysis

Results are presented as mean ± SEM. Significance was assessed using a two-tailed *P-*value calculated by the Mann-Whitney U test. *P*<0.05 is considered significant. All graphs were generated and statistical analyses performed using GraphPad Prism 8.0 software.

## Results

### Determine berberine concentration for BEAS-2B without significant cytotoxic effect

Before examining anti-inflammatory efficacy of berberine, we evaluated whether berberine has cytotoxicity on BEAS-2B cell line. Cells were pre-treated with different concentrations of berberine (0.1 μM-10 μM) or DMSO as control. Cells were stimulated with or without IL-4 plus TNF-α for activation and cell viability was assayed. The data indicated berberine at 1 μM or lower concentrations didn't affect the viability of BEAS-2B cells with or without pro-inflammatory cytokine stimulation compared with DMSO control (Figure 1B and C). Berberine had certain cytotoxic effect on BEAS-2B cells when treated with berberine higher than 10 μM (Figure 1 and data not shown). Corresponded concentrations of DMSO control to berberine had no toxicity on BEAS-2B cells. Thus, in subsequent experiments, we used 1 μM berberine to evaluate the anti-inflammatory effect of berberine on BEAS-2B cells.

### Berberine significantly reduced the production of IL-6 and CCL11 in pro-inflammatory cytokine-activated BEAS-2B cells

**Figure 2:**
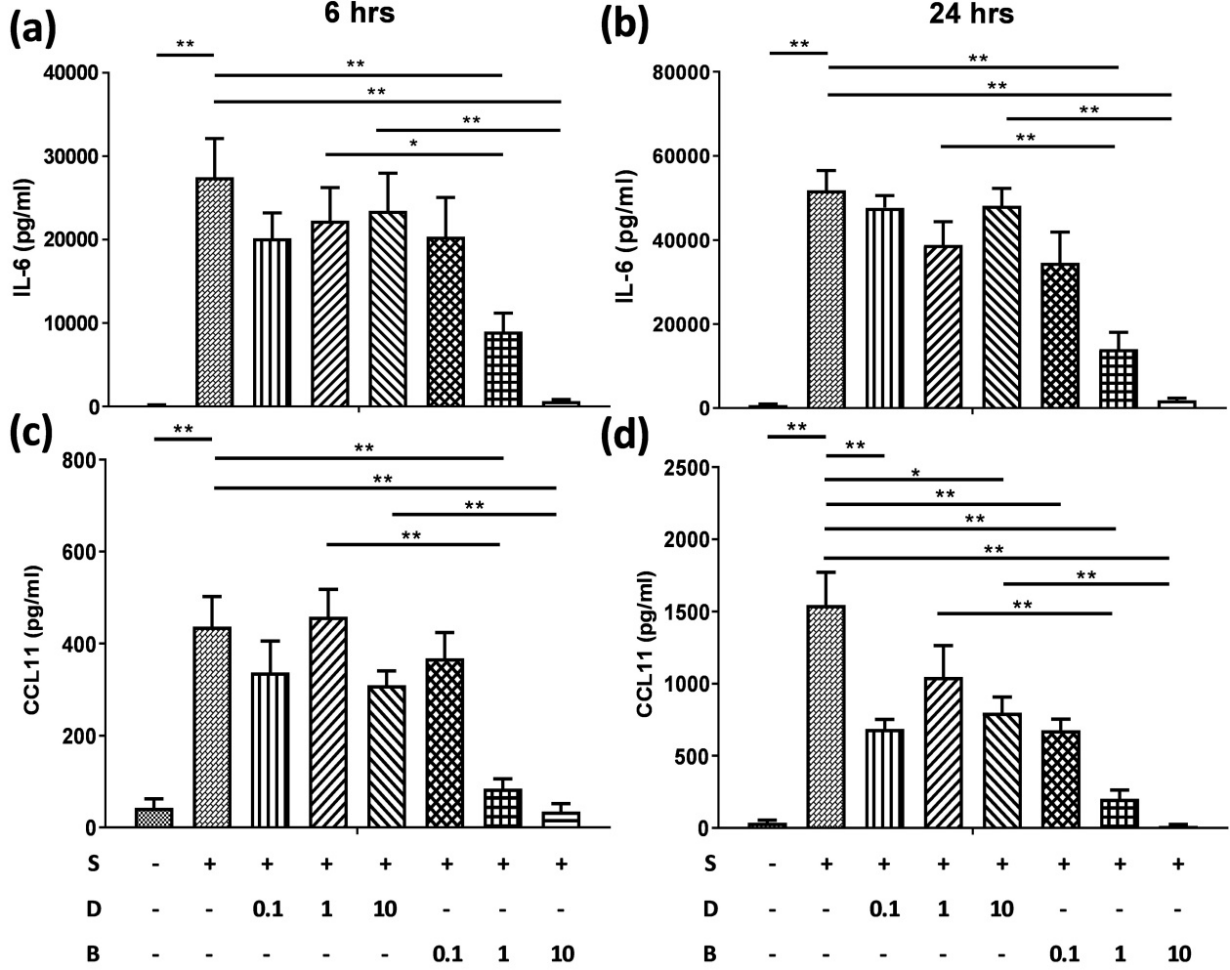
** Berberine pre-treatment suppressed pro-inflammatory cytokine-induced IL-6 and CCL11 production in the BEAS-2B cells.** BEAS-2B cells were seeded in the 48-well plate and treated with berberine (0.1 μM to 10 μM) or DMSO (control group) overnight. Cells with berberine pre-treatment were activated without or with IL-4 plus TNF-α for 6 and 24 hours. Culture supernatants were harvested and measured for (A, B) IL-6 and (C, D) CCL11 using ELISA. Data are presented as mean ± SEM (n= 6). S, pro-inflammation cytokine stimulation; D, DMSO; B, berberine. The number indicated the concentration (μM) of berberine or DMSO. *P<0.05; **P<0.01.

To assess the role of berberine on suppressing inflammatory mediators in bronchial epithelial cells, BEAS-2B cells were stimulated with IL-4 plus TNF-α for 6 and 24 hours. BEAS-2B cells without pro-inflammatory cytokine stimulation secreted low levels of IL-6 (Figure 2A and B) and CCL11 (Figure 2C and D). Significant amounts of IL-6 and CCL11 were detected in BEAS-2B cells after IL-4 plus TNF-α stimulation. Berberine (1 μM) significantly suppressed the protein levels of IL-6 (Figure 2A and B) and CCL11 (Figure 2C and D) in stimulated BEAS-2B cells compared to untreated group.

**Figure 3:**
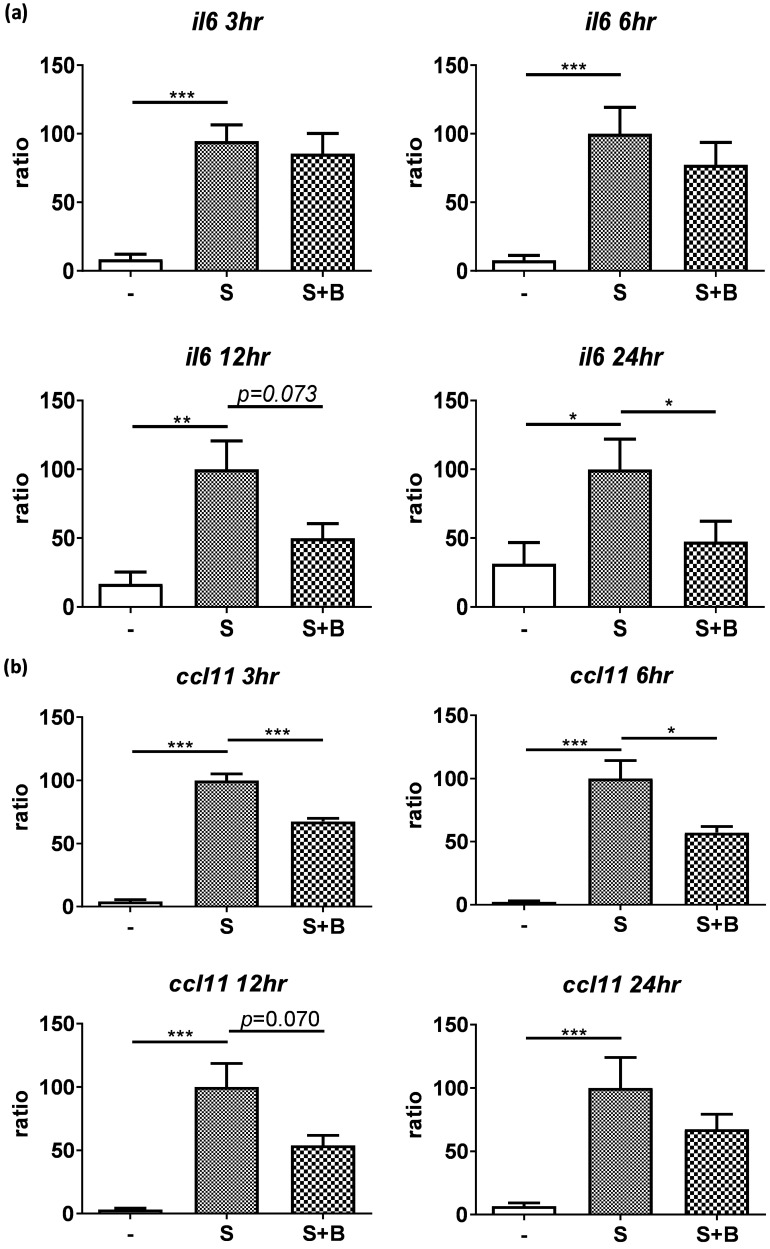
** Expression levels of IL-6 and CCL11 gene were reduced in pro-inflammatory cytokine-stimulated BEAS-2B cells with berberine pre-treatment. Total** RNA was extracted from IL-4 plus TNF-α-activated BEAS-2B cells pre-treated with berberine (1 μM) at 3, 6, 12, and 24 hours. The RNA expression levels of (A) IL-6 and (B) CCL11 detected by real-time PCR, normalized with GAPDH and compared with stimulation group (n=7). Data are presented as mean ± SEM. -, cell only; S, pro-inflammation cytokine stimulation; B, berberine. *P<0.05, **P<0.01, *** P<0.001.

To carefully examine the gene expression, total RNA were collected from stimulated BEAS-2B cells with berberine pre-treatment at different time points and the gene expression of IL-6 and CCL11 were analyzed by real-time PCR (Figure 3). Obvious RNA expression of IL-6 and CCL11 were detected in BEAS-2B cells with pro-inflammatory cytokine stimulation (Figure 3A and B). Berberine treatment reduced the expression levels of IL-6 and CCL11 genes in stimulated BEAS-2B cells, which is consistent with cytokine secretion pattern. These results indicated that reduced inflammatory mediators (IL-6 and CCL11) in response to berberine pre-treatment may alleviate activated BEAS-2B cells-mediated airway inflammation.

### Significant reduction of nuclear STAT6 protein expression in activated BEAS-2B cells with berberine treatment

**Figure 4:**
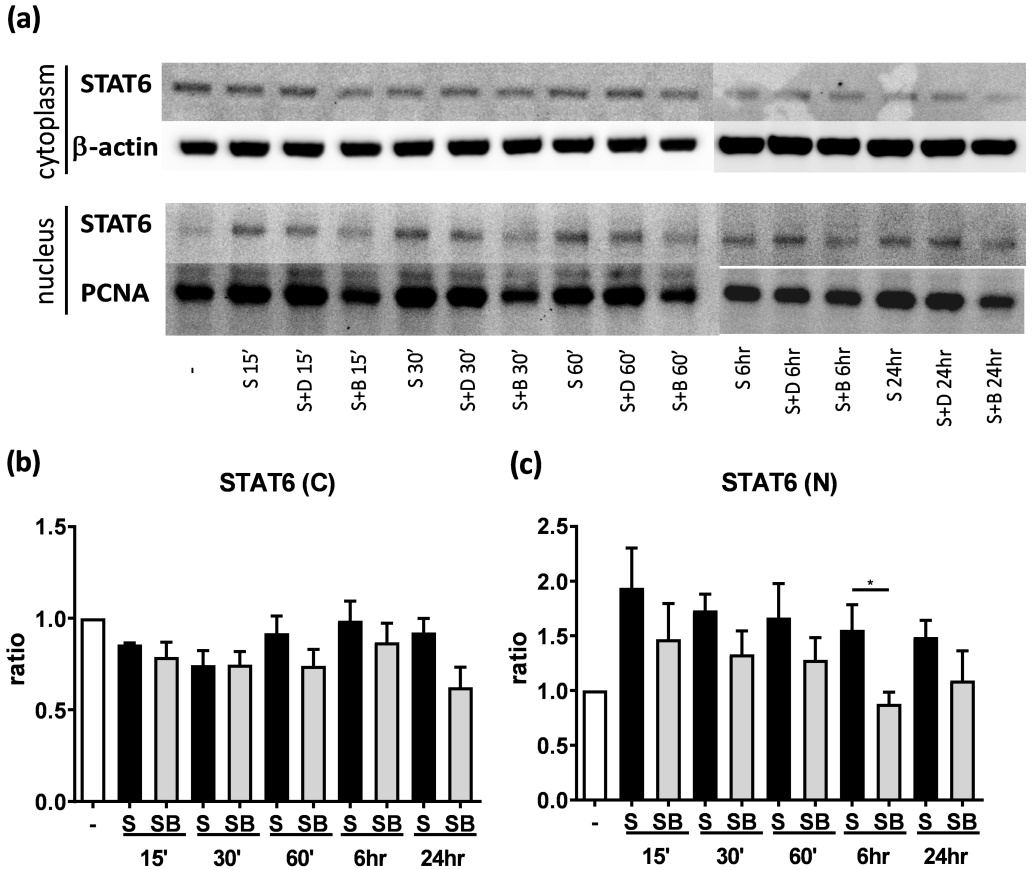
** Berberine down-regulates STAT6 protein expression in nucleus with pro-inflammatory cytokine stimulated BEAS-2B cells.** Cells with berberine (1 μM) treatment were harvested on the indicated time points after IL-4 plus TNF-α stimulation and cytoplasm and nucleus proteins were extracted. (A) Expression levels of cytoplasm and nucleus STAT6 proteins were analyzed using western blotting (20 μg per sample). β-actin and PCNA expression was used as an internal control. The relative quantity of (B) cytoplasm STAT6 (n=4) and (C) nucleus STAT6 (n=7) was normalized to β-actin and PCNA, respectively. Results are presented as mean ± SEM. *P<0.05. -, cell only; S, pro-inflammation cytokine stimulation; D, DMSO, B, berberine.

To investigate possible mechanisms involved in berberine-suppressed cytokine production, we first dissected the expression of STAT6 in BEAS-2B cells. Previous studies demonstrated that eotaxin-1 (CCL11) can be secreted from airway epithelial cells and fibroblasts after IL-4 plus TNF-α stimulation 17. The activation of STAT6 pathways play a pivotal role in regulating the expression of CCL11 gene 19. We analyzed cytoplasmic and nuclear STAT6 in activated cells with or without berberine treatment by western blotting (Figure 4A). In both cytoplasm (Figure 4B) and nucleus (Figure 4C), lower STAT6 protein expresion was observed after berberine treatment in activated BEAS-2B cells, while significantly decreased STAT6 protein was detected 6 hours after stimulation in the nucleus. Phosphorylated STAT6 expression was also decreased in nucleus after 60 minutes (Figure S1). These results suggest that berberine may reduce the expression of nuclear STAT6 protein in activated BEAS-2B cells to suppress the CCL11 production.

### Effect of berberine on MAP kinase pathways in activated BEAS-2B cells

**Figure 5:**
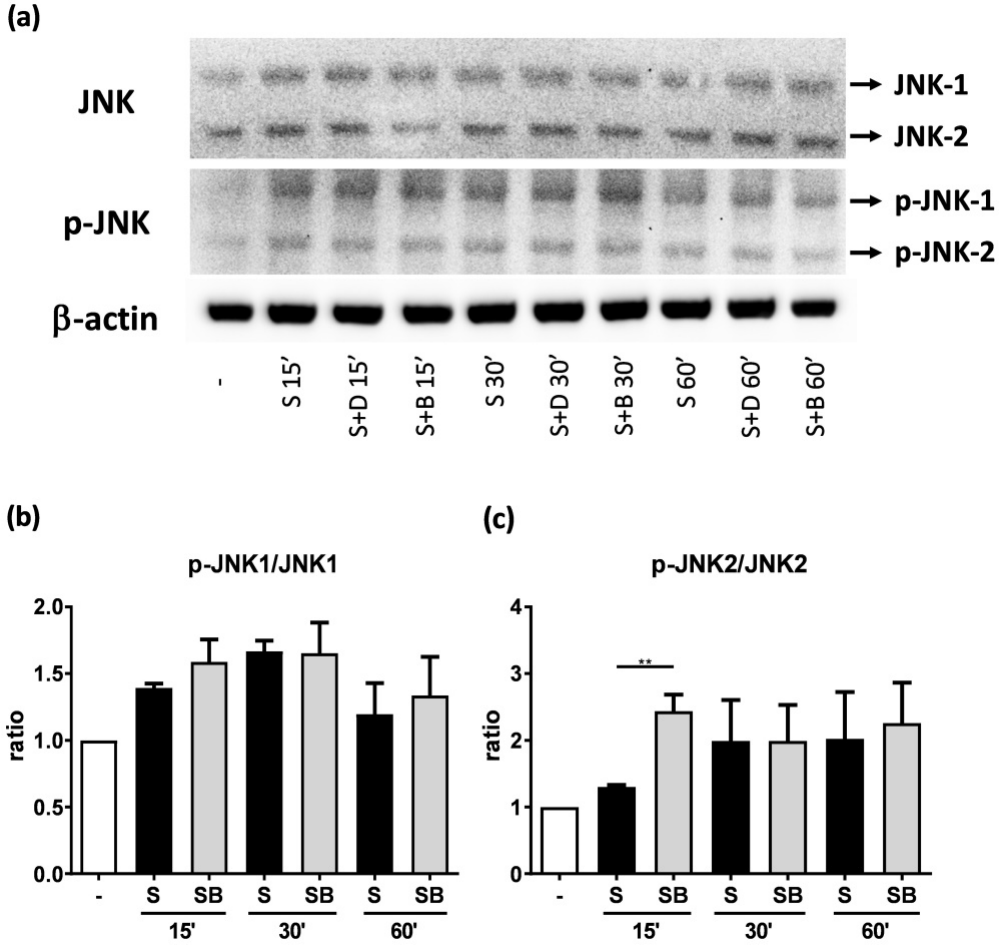
** JNK and p-JNK protein expression of activated BEAS-2B cells pre-treated with or without berberine.** Total cell proteins were collected at 15, 30, and 60 min of pro-inflammatory cytokine stimulation in BEAS-2B cells with berberine (1 μM) pre-treatment. (A) Expression levels of JNK and p-JNK proteins were analyzed using western blotting (20 μg per sample). The relative quantity of (B) p-JNK1 and (C) p-JNK2 was normalized to JNK1 and JNK2, respectively. β-actin expression was used as an internal control. Results are presented as mean ± SEM of six independent experiments. -, cell only; S, pro-inflammation cytokine stimulation; D, DMSO, B, berberine.

**Figure 6:**
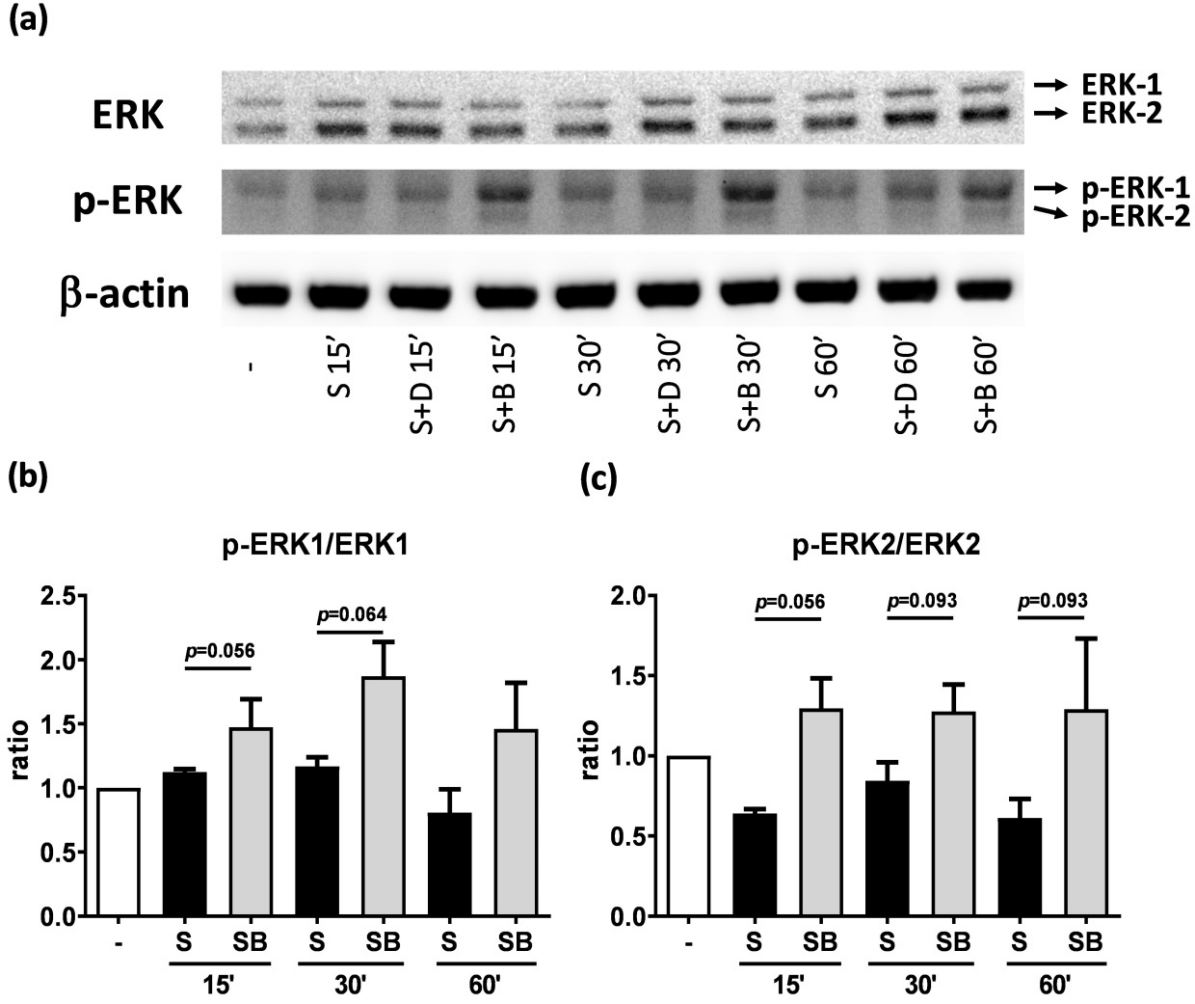
** Western blot analysis of ERK and p-ERK in pro-inflammatory cytokine stimulated BEAS-2B cells with berberine pre-treatment.** Total cell proteins were collected at 15, 30, and 60 min of IL-4 plus TNF-α stimulation in BEAS-2B cells with berberine (1 μM) pre-treatment. (A) Expression levels of ERK and p-ERK proteins were analyzed using western blotting (20 μg per sample). The relative quantity of (B) p-ERK1 and (C) p-ERK2 was normalized to ERK1 and ERK2, respectively. β-actin expression was used as an internal control. Results are presented as mean ± SEM of six independent experiments. -, cell only; S, pro-inflammation cytokine stimulation; D, DMSO, B, berberine.

**Figure 7:**
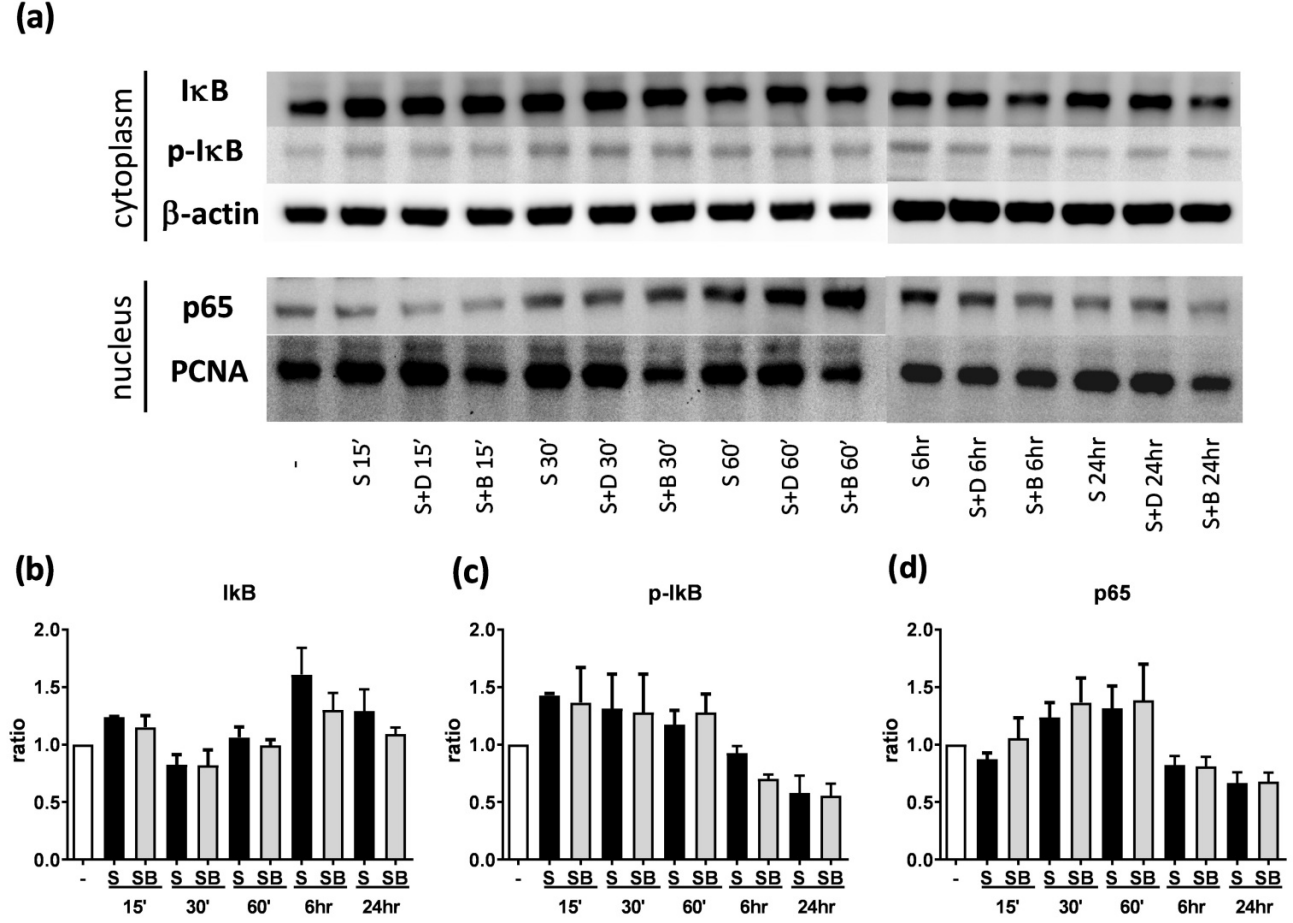
** Western blot analysis of IκB, p-IκB, and p65 in pro-inflammatory cytokine stimulated BEAS-2B cells with berberine pre-treatment.** Total cell proteins were collected at 15 min, 30 min, 60 min, 6 hours, and 24 hours of IL-4 plus TNF-α stimulation in BEAS-2B cells with berberine (1 μM) pre-treatment. (A) Expression levels of IκB, p-IκB, and p65 proteins were analyzed using western blotting (20 μg per sample). The relative quantity of (B) IκB (n=6), (C) p- IκB (n=3) and (D) nucleus p65 (n=6) was normalized to β-actin and PCNA, respectively. β-actin and PCNA expression was used as an internal control. Results are presented as mean ± SEM. -, cell only; S, pro-inflammation cytokine stimulation; D, DMSO, B, berberine.

To investigate how berberine suppressed IL-6 production in activated BEAS-2B cells, the expression and activity of MAP kinases and NF-kB were examined. The expression of p-JNK1/JNK1, p-JNK2/JNK2, p-ERK1/ERK1, and p-ERK2/ERK2 were detected in BEAS-2B cells with IL-4 plus TNF-α stimulation (Figure 5A and 6A). The expression levels of JNK and ERK proteins were not different between each group after stimulation (Figure 5A and 6A). Relative expression levels of p-JNK1/JNK1 remains no change after berberine treatment, while p-JNK2/JNK2 (Figure 5B) was elevated 15 minutes after stimulation but later was recovered (Figure 5C). On the other hand, p-ERK1/ERK1, and p-ERK2/ERK2 (Figure 6B and C) were elevated in activated BEAS-2B cells with berberine treatment compared to untreated groups, but did not show significant difference. No significant differences were detected in p38 protein expression in activated BEAS-2B cells with or without berberine treatment and p-p38 expression levels were undetectable among the different groups (data not shown). We confirmed this result by adding JNK inhibitor or ERK inhibitor into berberine-treated and cytokine-activated BEAS-2B cells. The level of IL-6 (Figure S2A) and CCL11 (Figure S2B) were still suppressed in berberine treated group after using JNK and ERK inhibitors. Thus, berberine did not obviously affect the MAP kinase pathways of activated BEAS-2B cells. Also, berberine did not influence the expression levels of IκB (Figure 7B), p-IκB (Figure 7C) or p65 (Figure 7D), a subunit of NF-κB, in activated BEAS-2B cells.

## Discussion

Allergic asthma is a chronic inflammatory disease of the airways that has become a global public health issue to result in a considerable burden on health services and expensive costs. The bronchial epithelium is recognized as a regulator of the initiation and maintenance of allergic airway inflammation 5. Despite a growing number of pharmaceutical and immunotherapy strategies for asthma, the development of effective and long-term immunomodulatory approach has been difficult. In many Asian countries, traditional Chinese medicine is routinely used as maintenance of daily health care or as complementary treatment for conventional Western medicine. Many evidences showed that traditional Chinese herb medicine has improved or therapeutic efficacy in allergic diseases, such as asthma, atopic dermatitis, and food allergy 27-29. Here, our results demonstrated that berberine is capable of suppressing the secretion of IL-6 and CCL11 in pro-inflammatory cytokine-activated BEAS-2B cells, human airway epithelial cells. The inhibition of pro-inflammatory cytokine-induced IL-6 and CCL11 production by berberine may cause by reduction of nuclear STAT6 expression in activated cells. Consequently, berberine may be applied as a candidate for the improvement or treatment of allergic asthma.

Corticosteroids and bronchodilators are the most common treatment of asthma 30. However, a few asthmatic patients respond poorly to these drugs or require higher doses to control the symptoms that lead to certain side-effects 31. Some patients seek to improve asthma symptoms using alternative medicine, including Chinese herbal medicine, yoga, homeopathy, and even urine therapy 32. Ma Xing Gan Shi Tang, Xiao Qing Long Tang, and Ding Chuan Tang are popular asthmatic complementary and alternative treatments which can modulate Th2 cell-driven airway inflammation 29, 33, 34. However, these herbal formulas contain *Ephedra spp.*, which contains ephedrine, causes excitement and unwanted side effects 35. Hence, other Chinese herbal formulas, single components, or pure compound extracts have been proved to relieve or treat the asthmatic symptoms, such as phloretin 36, ASHMI 37, MSSM-002 38, and CVT-E002 39. ASHMI, which is composed by *Sophora flavescens*, *Glycyrrhiza uralensis*, and *Ganoderma lucidum*, suppresses AHR, airway eosinophilia, and Th2 cell-secreted cytokines in asthmatic mice 37. ASHMI was also successfully applied for the Phase I of clinical trial to ameliorate FEV1, peak expiratory flow, and serum IgE levels 40. Extract from plants or compounds also have therapeutic effects on asthma. For example, extracts of *Nigella sativa* or purple passion fruit peel were also reported to reduce asthmatic symptoms like cough, wheeze, and shortness of breath in asthmatic patients 41, 42. Baicalin, a flavonoid compound isolated from *Scutellaria baicalensis,* was reported to suppress STAT3 expression and promotes FoxP3 expression to alleviate asthmatic symptoms in mice 43. Therefore, single components or pure compound extracts from various Chinese herbs are able to serve as an attractive approach to modulate allergic asthma.

Alkaloid-containing plants have been used as medicine for animal and human starting from 4000 years ago. Alkaloids and derived species have been widely used to treat a variety of illnesses 44. Berberine, an isoquinoline alkaloid that can be extracted from various Chinese herbs such as *Coptis chinensis*, *Hydrastis canadensis*, *Berberis aristata* and others, has potential of anti-inflammatory, anti-lipidemic, anti-neoplastic, and anti-diabetic activity 23, 45. A previous study showed that berberine (100 μM) was not toxic to A-549, U-937, and HFL-1 cell lines 24. Our results indicate that berberine has dose-dependent cytotoxicity in BEAS-2B cells, although berberine at lower than 1 μM is innocuous. At this concentration, berberine significantly reduced the secretion of IL-6 and CCL11 in IL-4 plus TNF-α-activated BEAS-2B cells. The activation of eotaxin-1 gene expression in IL-4 plus TNF-α-stimulated airway epithelial cells and fibroblasts was regulated by activating JAK1/3-STAT6 pathway 19. After phosphorylation, STAT6 form homodimers and enter the nucleus 46. A report has demonstrated that berberine inhibits IL-2 induced JAK3 phosphorylation in monoarhritic rats 47. In our results, berberine significantly repressed the expression of nuclear STAT6 in activated BEAS-2B cells and reduces CCL11 levels. In ovalbumin (OVA)-induced rat model of asthma, berberine has been reported to relieve inflammatory cell infiltration, lung inflammation, and IgE production 48. The suppressive effects on the airway inflammation might be mediated through the inhibition of NF-κB signaling pathway by berberine treatment. Berberine blocks the caspase1/NF-κB pathway to reduce thymic stromal lymphopoietin (TSLP) production in human mast cell line, HMC-1 cells 49. However, in activated BEAS-2B cells, berberine didn't decrease the expression levels of IκB and NF-κB (p65 subunit). Berberine was also proposed to induce the production of IL-12 p40 by activating p38 MAP kinase in mouse macrophages 50. In ARPE-19 cells, IL-6 secretion was stimulated by TNF-a through p38 MAP kinase, while berberine down-regulated the phosphorylation of p38 MAP kinase 26. Although JNK and ERK might be elevated after berberine treatment, we demonstrated that the decrease of IL-6 and CCL11 were not caused by JNK or ERK activation by adding MAP kinase inhibitors. Thus, whether berberine is able to ameliorate the asthmatic symptoms by reducing IL-6 and CCL11 secretion of airway epithelial cells *in vivo* is needed to further investigate. In conclusion, berberine perhaps is able to relieve airway inflammation by suppressing cytokine and chemokine production of epithelial cells.

## Supplementary Material

Supplementary figures and tables.

## Abbreviations

Th: T helper
IL: Interleukin
AHR: Airway hyperresponsiveness
EBC: Exhaled breath condensate
TNF: Tumor necrosis factor
MAPK: Mitogen-activated protein kinase
NF-κB: Nuclear factor-kappa B
IκB: Inhibitor of kappa B
JNK: c-Jun NH2-terminal kinase
ERK: Extracellular signal-regulated kinase
DMSO: Dimethyl sulfoxide
CCK-8: Cell Counting-kit 8
TSLP: Thymic stromal lymphopoietin
OVA: Ovalbumin
