# Supplementary material for: Berberine Inhibits Pro-inflammatory Cytokine-induced IL-6 and CCL11 Production via Modulation of STAT6 Pathway in Human Bronchial Epithelial Cells
Source: Int J Med Sci. 2020 Jun 8;17(10):1464–73. doi: 10.7150/ijms.45400 (PMC7330667; doi:10.7150/ijms.45400)
Supplement: Supplementary file 1 — Supplementary figures and tables. [file ijmsv17p1464s1.pdf]

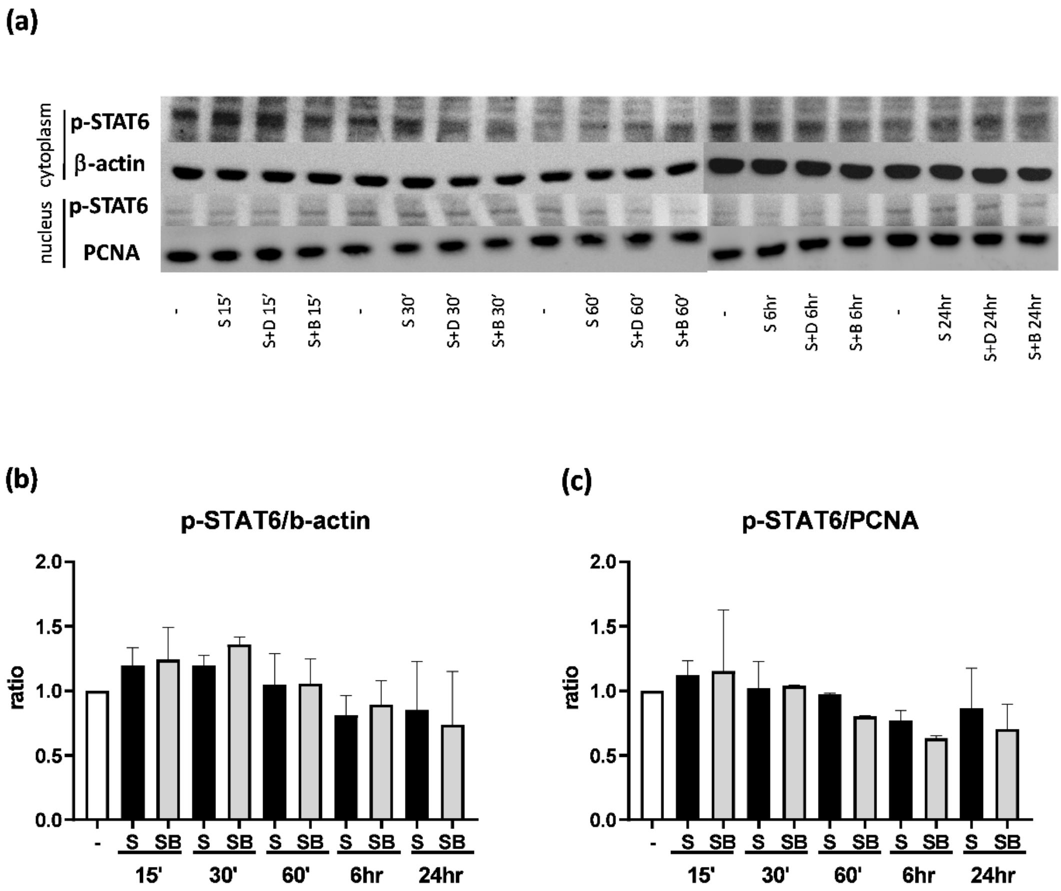

Fig. S1 Berberine down-regulates p-STAT6 protein expression in nucleus with pro-inflammatory cytokine stimulated BEAS-2B cells. Cells with berberine (1 μM) treatment were harvested on the indicated time points after IL-4 plus TNF-α stimulation and cytoplasm and nucleus proteins were extracted. (A) Expression levels of cytoplasm and nucleus p-STAT6 proteins were analyzed using western blotting (20 μg per sample). β-actin and PCNA expression was used as an internal control. The relative quantity of (B) cytoplasm p-STAT6 (n=2) and (C) nucleus p-STAT6 (n=2) was normalized to β-actin and PCNA, respectively. Results are presented as mean ± SEM. -, cell only; S, pro-inflammation cytokine stimulation; D, DMSO, B, berberine.

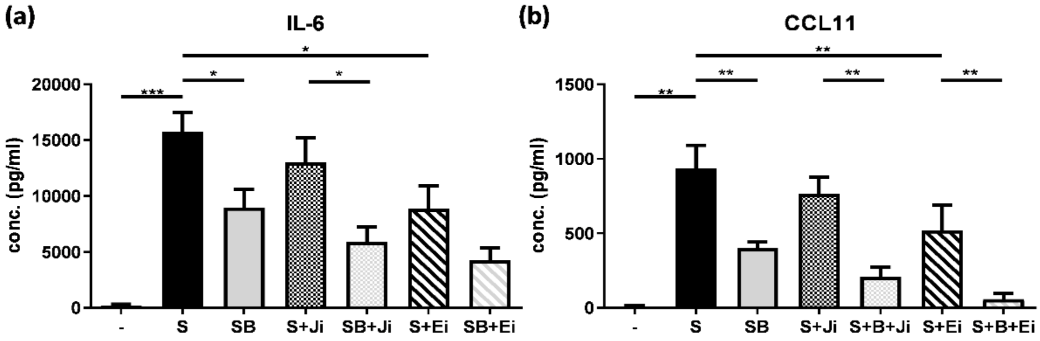

Fig. S2 MAP kinase inhibitors pre-treatment did not affect the reducing pattern of pro-inflammatory cytokine-induced IL-6 and CCL11 production in berberine pre-treatment BEAS-2B cells. BEAS-2B cells were seeded in the 48-well plate and treated with berberine (1 μM) overnight. Cells with berberine pre-treatment were activated without or with IL-4 plus TNF-α for 24 hours. Inhibitors (SP600125 or PD-98059 10 μM) were added one hour before IL-4 plus TNF-α stimulation. Culture supernatants were harvested and measured for (A) IL-6 and (B) CCL11 using ELISA. Data are presented as mean ± SEM (n=6). S, pro-inflammation cytokine stimulation; B, berberine; Ji, JNK inhibitor SP600125; Ei, ERK inhibitor PD-98059. *$P<0.05$; **$P<0.01$, ***$P<0.001$.
